# Prolylcarboxypeptidase promotes IGF1R/HER3 signaling and is a potential target to improve endocrine therapy response in estrogen receptor positive breast cancer

**DOI:** 10.1080/15384047.2022.2142008

**Published:** 2022-11-04

**Authors:** Lei Duan, Sarah J. Calhoun, Ricardo E. Perez, Virgilia Macias, Fatima Mir, Paolo Gattuso, Carl G. Maki

**Affiliations:** a Department of Anatomy and Cell biology, Rush University Medical Center, Chicago, IL, 60612, USA; b Department of Pathology, University of Illinois at Chicago, Chicago, IL, 60612, USA; c Department of Pathology, Rush University Medical Center, Chicago, IL, 60612, USA

**Keywords:** PRCP, IGF1R, HER3, endocrine therapy, prognosis

## Abstract

Prolylcarboxypeptidase (PRCP) is a lysosomal serine protease that cleaves peptide substrates when the penultimate amino acid is proline. Previous studies have linked PRCP to blood-pressure and appetite control through its ability to cleave peptide substrates such as angiotensin II and α-MSH. A potential role for PRCP in cancer has to date not been widely appreciated. Endocrine therapy resistance in breast cancer is an enduring clinical problem mediated in part by aberrant receptor tyrosine kinase (RTK) signaling. We previously found PRCP overexpression promoted 4-hydroxytamoxifen (4-OHT) resistance in estrogen receptor-positive (ER+) breast cancer cells. Currently, we tested the potential association between PRCP with breast cancer patient outcome and RTK signaling, and tumor responsiveness to endocrine therapy. We found high PRCP protein levels in ER+ breast tumors associates with worse outcome and earlier recurrence in breast cancer patients, including patients treated with TAM. We found a PRCP specific inhibitor (PRCPi) enhanced the response of ER+ PDX tumors and MCF7 tumors to endoxifen, an active metabolite of TAM in mice. We found PRCP increased IGF1R/HER3 signaling and AKT activation in ER+ breast cancer cells that was blocked by PRCPi. Thus, PRCP is an adverse prognostic marker in breast cancer and a potential target to improve endocrine therapy in ER+ breast cancers.

## Introduction

Endocrine therapy is standard care for women with estrogen receptor positive (ER+) breast cancer. Tamoxifen (TAM) is a selective estrogen receptor modulator (SERM) that binds ER and antagonizes ER signaling. TAM is used to treat both pre- and post-menopausal women whose breast cancers are ER+,^1,2^ although it is slightly inferior in efficacy for post-menopausal women compared to aromatase inhibitors. Aromatase inhibitors (AIs) lower estrogen levels by blocking the conversion of androgen to estrogen by the enzyme aromatase, and are used for treatment of post-menopausal women with ER+ breast cancer.^3–5^ TAM and AIs have been mainstay treatment options for many years and have improved outcomes in breast cancer patients. Nonetheless, resistance to these endocrine therapies can and do develop, which can lead to tumor recurrence and poor patient outcomes.^6,7^ An important goal is to identify factors that promote TAM/endocrine therapy resistance and ways to target them.

In our previous study, we identified Prolylcarboxypeptidase (PRCP) as a tamoxifen resistance factor.^8^ The PI3K-AKT pathway is activated downstream of multiple different receptor tyrosine kinases (RTKs) and can promote cancer cell survival as well as resistance to endocrine therapies.^9–12^ Overexpression of PRCP induced 4-OHT resistance in ER+ MCF7 breast cancer cells.^8^ Though the mechanism by which PRCP promotes 4-OHT resistance is unknown, it is important to note that in other studies we found PRCP maintains PI3K and AKT activation in pancreatic cancer cells.^13^ Thus, though it has not yet been investigated, we speculate PRCP may promote signaling downstream of RTKs in ER+ breast cancers in order to maintain PI3K-AKT activation and promote 4-OHT resistance. The current study was undertaken to address three questions: 1) what is the relationship between PRCP expression and breast cancer patient outcome, including patients treated with endocrine therapy? 2) Does PRCP promote RTK signaling in breast cancer cells as a possible mechanism of endocrine therapy resistance? 3) Is PRCP a potential target to enhance endocrine therapy response in ER+ breast cancer?

## Results

### High expression of PRCP protein in breast cancers associated with worse outcome

**Figure 1.**
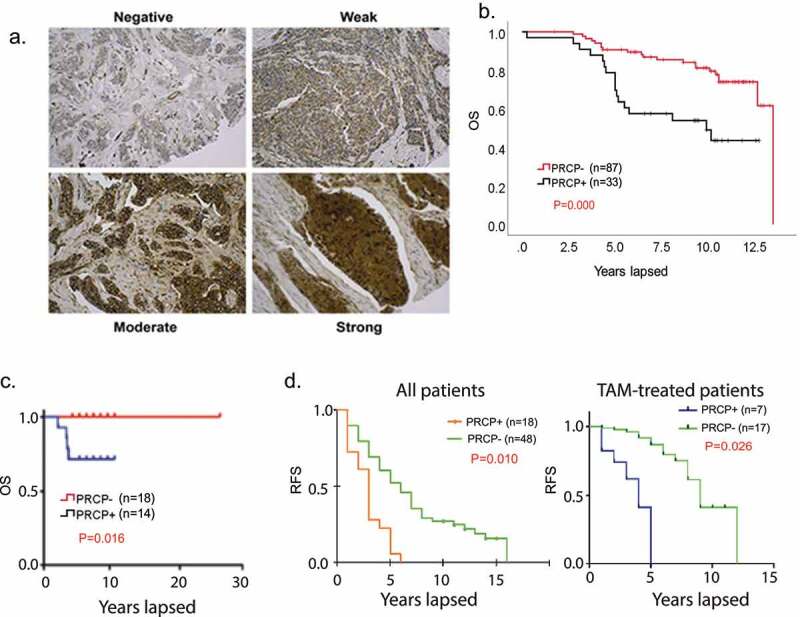
PRCP-positive BC patients have poor outcomes. A. Slides containing cores of breast cancer tissues were deparaffinized and stained with primary PRCP antibody (# HPA017065, Human Protein Atlas). PRCP displayed a granular cytoplasmic pattern. TMA staining was scored based on intensity (negative, weak, moderate, and strong) and percent cell staining. The criteria for PRCP positivity are that more than 60% of the cells have moderate to strong staining. B. Primary breast cancer tissues from 120 ER+/Her2- BC patients treated at Rush Medical Center from 2000–2005 were stained for PRCP. Kaplan-Meier curves show overall survival (OS) is significantly lower (P = .000) in PRCP positive (N = 33) patients compared with PRCP negative patients (N = 87). C. Primary breast cancer tissues of 32 ER+/Her2- BC patients (Fox Chase TMA) were stained for PRCP. Kaplan-Meier shows OS is significantly shorter (P = .016) in PRCP positive patients (N = 14) compared with PRCP negative patients (N = 18). D. Left panel: primary breast cancer tissues of 66 recurrent ER+/Her2- BC patients treated at UIC from 1994–2004 were stained for PRCP. Kaplan-Meier shows RFS is significantly shorter (P = .010) in PRCP positive patients (N = 18) compared with PRCP negative patients (N = 48). Right panel: among the 66 patients, 24 patients were treated with tamoxifen. Kaplan-Meier shows RFS is significantly shorter (P = .026) in PRCP positive patients (N = 7) compared with PRCP negative patients (N = 17).

The first goal of this study was to address whether and how PRCP expression relates to breast cancer patient outcome, including patients treated with endocrine therapy. To that end, we first created a tissue microarray (TMA) from 120 estrogen receptor positive (ER+/Her2-) breast cancer patients treated at our institute from 2000 to 2005 and for whom long-term survival data is available. Representative staining patterns are shown in Figure 1a. The criteria for PRCP positivity was that more than 60% of cells have moderate to strong PRCP staining. The results showed PRCP positivity associates with significantly reduced overall survival in these patients (Figure 1b). We carried out a similar analysis using a 32 breast cancer patient TMA from Fox Chase Cancer Center (Figure 1C). The data also showed high PRCP expression associates with reduced overall survival. Among the 152 patients from both TMAs, 124 of them have complete record of clinical stages and node status. Stage distribution related to PRCP positivity is listed in Table 1. We further carried out multivariate analysis for correlation of OS with stage, node positivity and PRCP positivity. The results showed that OS significantly correlates stage and PRCP positivity but not with node positivity (Table 2). Further, in stage I and Stage II patients OS correlates with PRCP positivity but not with node positivity (Tables 3 and 4). Lastly, we analyzed association between PRCP positivity and tumor recurrence in a 66 patient TMA from recurrent breast cancer patients. The results showed PRCP positivity correlates with reduced RFS (earlier recurrence) in all patients, including patients treated with TAM (Figure 1d). Overall, the results in Figure 1 indicate high PRCP protein expression is associated with worse outcome and earlier recurrence in breast cancer patients, including in patients treated with endocrine therapy.

**Table 1.** PRCP positivity according to the stages.

Stage	PRCP positive	PRCP negative
Stage I (n = 77)	21 (27.3%)	56 (72.7%)
Stage II (n = 38)	13 (34.2%)	25 (65.8%)
Stage III (n = 5)	2 (40%)	3 (60%)
Stage IV (n = 4)	4 (100%)	0 (0%)

**Table 2.** Multivariate analyses for overall survival in all cases (Cox regression model). (RR, relative risk); C.I., confidence interval; PRCP, prolylcarboxypeptidase).

All patients			
Variables	RR	95% C.I.	*p*
Stage (number)	0.657	1.168–3.183	0.01
Nodes (number)	0.386	0.792–2.734	0.222
PRCP positivity	1.43	1.843–9.484	0.001

**Table 3.** Multivariate analyses for overall survival in stage I cases (Cox regression model).

Stage I patients			
Variables	RR	95% C.I.	*p*
Nodes (number)	−0.519	0.076–4.660	0.621
PRCP positivity	1.321	1.407–9.980	0.008

**Table 4.** Multivariate analyses for overall survival in stage II cases (Cox regression model).

Stage II patients			
Variables	RR	95% C.I.	*p*
Nodes (number)	0.25	0.709–2.324	0.409
PRCP positivity	1.595	1.129–21.539	0.034

### Overexpression of PRCP increases AKT-mTORC1 and IGF1R/HER3 signaling

**Figure 2.**
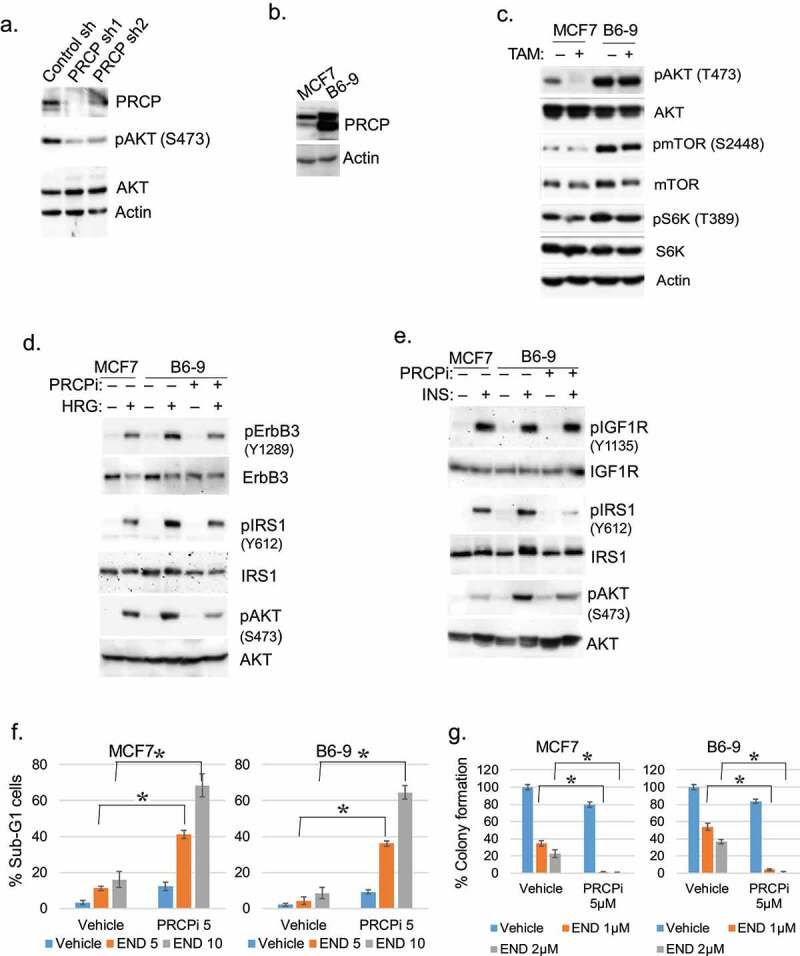
PRCP promotes ErbB3/IGFR/IRS1/AKT activation. A. Lysates of MCF7 cells infected with control shRNA of PRCP shRNA (#1 and #2) were immunoblotted for the indicated proteins. B. Lysates of MCF7 and B6-9 cells were immunoblotted for the indicated proteins. C. MCF7 and B-9 cells were treated with 4OHTAM (10 μM) for 24 h. Lysates were immunoblotted for the indicated proteins. Serum starved MCF7 and B6-9 cells were pretreated with PRCPi (10 μM) and then treated with heregulin (HRG) (d) or insulin (INS) (e) for 10 min. Lysates were immunoblotted for the indicated proteins. All immunoblots are representative images of two-three independent experiments. F. The cells were treated with Endoxifen (END, 5 µM and 10 μM) and/or PRCPi (5 µM) for 72 h and then analyzed with FACS for cell cycle. Average (triplicate) % sub-G1 cells were presented with SD indicated. There are significant differences (P < .01) between PRCPi+END and END (both doses) groups. G. The cells were treated with Endoxifen (END, 1 µM and 2 μM) and/or PRCPi (5 µM) for 72 h and then grown in drug free media for 4 weeks until colony formed. Average (triplicate) % colony formation is presented with SD indicated. There are significant differences (P < .01) between PRCPi+END and END (both doses) groups.

Aberrant RTK signaling contributes to endocrine therapy resistance in breast cancer. AKT and mTORC1 are activated downstream of RTKs and promote endocrine therapy resistance.^14–16^ We reported PRCP maintains levels of activated AKT (S473 phosphorylated) in pancreatic cancer cells.^13^ Therefore, we hypothesized PRCP may promote endocrine therapy resistance by regulating RTK signaling, including activation of AKT. To examine this, we first tested the effect of PRCP knockdown in ER+ MCF7 breast cancer cells using two different shRNAs. The results showed PRCP knockdown reduced levels of activated (S473 phosphorylated) AKT in MCF7 cells (Figure 2a). We also tested the effect of PRCP overexpression. B6-9 cells are 4-OHT-resistant MCF7 cell derivatives that stably over-express PRCP (Figure 2b). As shown in Figure 2c, B69 cells expressed higher basal levels of activated (S473 phosphorylated) AKT compared to MCF7 cells and maintained activated AKT levels after TAM treatment, whereas in MCF7 cells activated AKT was lost in response to 4-OHT. mTORC1 was also more activated in PRCP overexpressing B69 cells compared to MCF7 cells, evidenced by increased mTOR phosphorylation at S2448 (autophosphorylation site) and increased S6K phosphorylation at T389 (mTORC1 substrate). Next, we examined the effect of PRCP overexpression or inhibition on signaling downstream of individual RTKs. Overexpression or activation of RTKs such as EGFR, HER2 and IGF1R can cause resistance to tamoxifen through activation of PI3K/AKT and MAPK pathways in breast cancer (BC) cell lines^14–16^ and are associated with poor outcome in tamoxifen-treated patients.^17,18^ As MCF7 cells express very low levels of EGFR/HER2 but high levels of HER3 (data not shown), we used heregulin (HRG) to activate HER3 in MCF7 and B6-9 cells. We found B6-9 cells had higher Y1289 phosphorylation (activation) of ErbB3 in response to the ErbB3 ligand HRG than MCF7 cells (Figure 2d). B6-9 cells also showed higher phosphorylation (activation) of IRS1 (Y612) and AKT (S473) than MCF7 cells in response to both HRG and insulin (INS) (Figure 2d and 2e). Importantly, co-treatment of B6-9 cells with a PRCP inhibitor PRCP-7414 (PRCPi) blocked or reduced ErbB3, IRS1, and AKT activation in response to HRG or INS (Figure 2d and 2e). We previously reported that B6-9 cells are less sensitive to 4OHTAM compared with MCF7 cells.^8^ We tested if PRCPi can sensitize MCF7 and B6-9 cells to endoxifen, the primary active metabolite of TAM. The cells were treated with endoxifen and/or PRCPi for 72 h and then either immediately assessed for the percent cells with sub-G1 DNA content as an indicator of cell death, or continued to grow in the absence of drug and assessed for long-term colony forming ability. As shown in Figure 2f and 2g, B6-9 cells were less susceptible to endoxifen-induced death compared with MCF7 cells in both assays, supporting that PRCP overexpression increases endoxifen resistance. PRCPi alone (5 μM) induced a slight increase in death in both MCF7 and B6-9 cells. However, combination of PRCPi with endoxifen induced a significant increase in cell death as determined by both % sub-G1 cells and colony formation (Figure 2f and 2g). The results support that PRCP promotes endocrine therapy (endoxifen) resistance and this is associated with heightened IGF1R/HER3 signaling.

### PRCP gene expression correlates with IGF1 and NRG1 expression and earlier recurrence of endocrine therapy treated breast cancer patients

**Figure 3.**
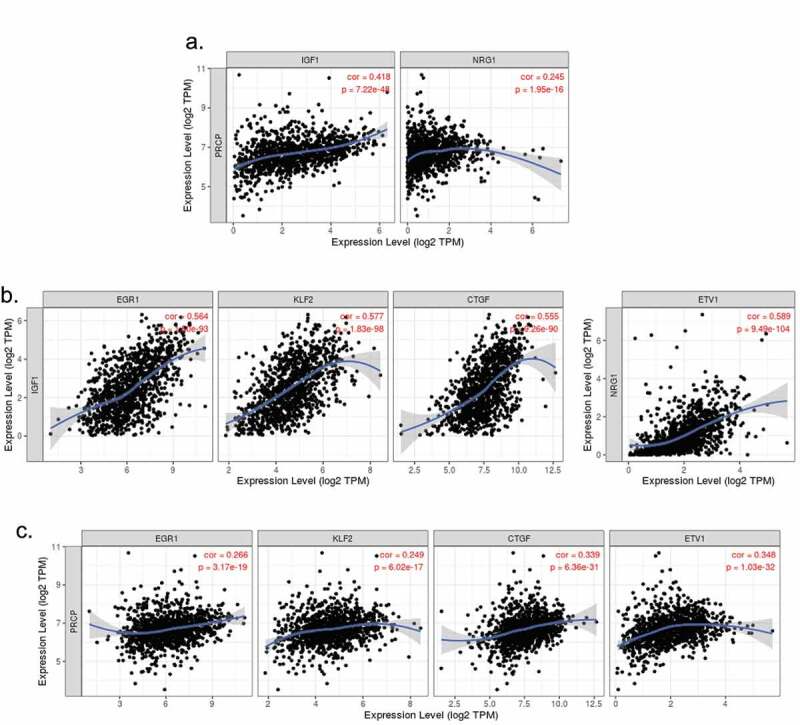
PRCP gene correlates with IGF1/NRG1 genes and their target genes in invasive breast cancer. TIMER analysis of gene correlation in 1093 cases of invasive breast cancer showed that PRCP positively correlates with IGF1 and NRG1 (a). IGF1 positively correlates with EGR1, KLF2, and CTGF and NRG1 positively correlates with ETV1 (b). PRCP positively correlates with EGR1, KLF2, CTGF, and ETV1 (c). Correlation coefficient and p values are indicated on the graphs.

**Figure 4.**
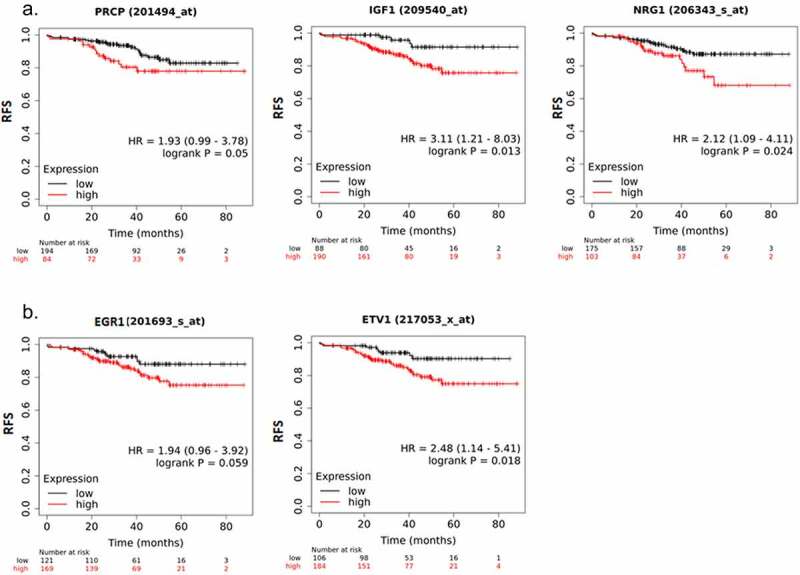
High expression of PRCP, IGF1/NRG1 and target genes correlates with poor prognosis. A. Analysis of PRCP, IGF1, and NRG1 gene expression in the GSE25066 dataset for ER+/HER2- BC patients treated with endocrine therapy. Kaplan-Meier shows that RFS is significantly shorter in *PRCP* high, *IGF1* high, and *NRG1* high patients compared with gene expression low patients. B. The IGF1 target genes *EGR1* (*AT225*) and the NRG1 target gene *ETV1* also significantly correlates with reduced RFS in the patients. P values (log-rank test) and case numbers are indicated on the graphs.

The above results suggest PRCP promotes IGF1R/HER3 signaling in ER+ breast cancer cells. We sought to investigate if PRCP promotes IGF1R/HER3 signaling in clinical patients. First, we analyzed correlation between PRCP gene expression and IGF1 (IGF1R ligand) and NRG1 (HER3 ligand) expression (Figure 3a) in 1093 cases of invasive breast cancer (TCGA database) using TIMER software. The results showed that *PRCP* positively correlated with *IGF1* and *NRG1* (Figure 3a, p values indicate significance). *EGR1, KLF2*, and *CTGF* are target genes activated downstream of IGF1.^19^ *ETV1* is a target gene activated downstream of NRG1 signaling.^20^ We found that *IGF1* positively correlates with *EGR1, KLF2*, and *CTGF* and *NRG1* positively correlates with *ETV1* with high significance (Figure 3b). *PRCP* also positively correlated with *EGR1, KLF2, CTGF, and ETV1* (Figure 3c. p values indicate significance). These results suggest that high expression of PRCP coincides with heightened IGF1/HER3 signaling in clinical patients. Next, we analyzed the GSE25066 dataset that contains 290 ER+/HER2- breast cancer patients treated with endocrine therapy.^21,22^ Kaplan-Meier survival curve analysis showed that high expression of *PRCP, IGF1* and *NRG1* significantly correlates with decreased RFS in the endocrine therapy treated patients (Figure 4a). High expression of *EGR1* and *ETV1* also significantly correlates with decreased RFS in these patients (Figure 4b). Altogether, these results indicate that high expression of PRCP correlates with increased IGF1R/HER3 signaling and endocrine therapy resistance in clinical patients.

### PRCP inhibitor combined with endoxifen causes regression of ER+ breast cancer tumors in mice

**Figure 5.**
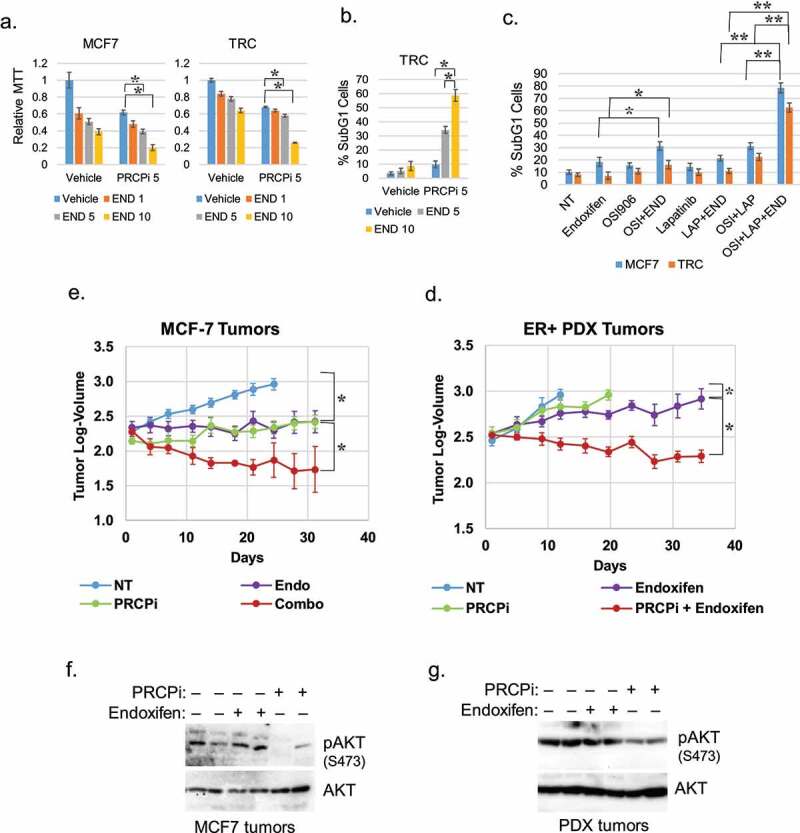
PRCPi sensitizes cells and tumors to endoxifen. MCF7 and TRC cells were treated with endoxifen and/or PRCPi for three days. Cells were analyzed with MTT assay or FACS for sub-G1. Average (8 replicates) relative MTT absorbance (a) and Average (triplicate) % sub-G1 cells (b) are presented with SD indicated. There are significant differences between PRCPi and PRCPi plus 5 µM and 10 µM endoxifen (p ˂ 0.05) in MCF7 and TRC cells in A. There are significant differences between PRCPi and PRCPi plus 5 µM and 10 µM endoxifen (p,0.01) in TRC cells in B. C. MCF7 and TRC cells were treated with endoxifen and/or OSI906 (5 µM) or lapatinib (2 µM) for three days. Cells were analyzed with FACS for sub-G1. Average (triplicate) % sub-G1 cells are presented with SD indicated. There are significant differences (p ˂ 0.05) between NT and endoxifen, endoxifen and endoxifen plus OSI906, OSI906 and OSI906 plus endoxifen, NT and OSI906 plus lapatinib, OSI906 plus lapatinib and endoxifen plus OSI906 plus lapatinib in MCF7 cells. There are no significant differences (p ˃ 0.05) between NT and endoxifen, endoxifen and endoxifen plus OSI906, OSI906 and OSI906 plus endoxifen, endoxifen and endoxifen plus lapatinib in TRC cells. There are significant differences (p˂0.01) between NT and OSI906 plus lapatinib, between OSI906 plus lapatinib and OSI906 plus lapatinib plus endoxifen. D and E. MCF7 tumors and ER+ PDX tumors were treated with vehicle. Endoxifen, PRCPi, or combination for the indicated times. Log-tumor volumes are plotted with SE indicated. The original tumor volume in mm^3^ for this data is presented in Fig. S1A and B. There are significant differences between vehicle and endoxifen or PRCPi (P < .05) in both tumors. There are significant differences between single drug and combination therapies (P < .05) in both tumors. F and G. At necropsy, tumors were harvested. Lysates were immunoblotted for the indicated proteins.

Next, we wished to ask if PRCP inhibition is effective against ER+ breast cells and tumors when given alone, or in combination with endocrine therapy. TRC cells are TAM-resistant derivatives of MCF7 cells that were generated by prolonged exposure to TAM.^8^ First, we treated MCF7 and TRC cells with increasing doses of endoxifen alone or in combination with PRCP inhibitor (5 μM). We monitored proliferation by MTT assay and cell death by the percentage of cells with sub-G1 DNA content. Combination of PRCPi plus endoxifen caused a pronounced inhibition of proliferation by MTT assay in both MCF7 and TRC cells (Figure 5a) and a pronounced increase in cell death by sub-G1 cells in TRC cells (Figure 5b). The results indicate PRCP inhibitor can increase endoxifen sensitivity in ER+ and TAM resistant cells. Next, we tested if inhibition of IGF1R and HER3 sensitizes MCF7 and TRC cells to endoxifen. OSI906 is an IGF1R inhibitor. HER3 does not have an active kinase domain so we used lapatinib that inhibits EGFR and HER2 that are HER3 dimerization partners. It is noteworthy that lapatinib blocks HRG-induced phosphorylation of HER3 in MCF7 cells (data not shown). OSI906 alone modestly sensitized MCF7 cells but not TRC cells to endoxifen while lapatinib alone did not sensitize MCF7 or TRC cells to endoxifen (Figure 5c). However, combined treatment with OSI906 and lapatinib significantly sensitized both MCF7 and TRC cells to endoxifen (Figure 5c). Lastly, we established ER+ MCF7 cell tumors and an ER+ human breast PDX tumor in the mammary fat pads of NSG mice. Tumor-bearing mice were then treated with vehicle, PRCPi alone, endoxifen alone, or both, and tumor growth monitored over 5 weeks. Data from this experiment is presented in Figure 5d and 5e as tumor log-volume, and the same data is presented in Figs S1A and B as tumor volume in mm^3^. The results showed that both endoxifen alone and PRCPi alone blocked or slowed the growth of MCF7 and PDX tumors. Strikingly, however, combined treatment with PRCPi and endoxifen caused a significant regression of the MCF7 and PDX tumors. Notably, mice appeared to tolerate the drug combination treatment without obvious weight loss (Figure S1). Immunoblots of tumor lysates showed pAKT (S473) was reduced by PRCPi compared with vehicle or endoxifen treated tumors (Figure 5f and 5g). These results suggest PRCPi is effective in vivo in suppressing tumor growth as a single drug and can synergistically induce regression of ER+ tumors when combined with endoxifen.

## Discussion

PRCP is a serine protease that localizes mainly in the lysosome but can also be extracellular.^23^ PRCP is known to regulate blood pressure and appetite control through its cleavage of peptide substrates angiotensin II and α-MSH.^24–26^ A role for PRCP in breast cancer or other cancers has, to date, not been widely recognized. In the current report, we found high PRCP protein expression associates with worse outcome and earlier recurrence in breast cancer patients, including ER+ patients treated with TAM. In addition, we found that high expression of PRCP correlates with increased expression of IGF1/NRG1 and their target genes and earlier recurrence of endocrine treated ER+ breast cancer patients. Overexpression of PRCP increased IGF1R/HER3 signaling and AKT-mTORC1 activation in ER+ breast cancer cells. A small-molecule PRCP inhibitor blocked IGF1R/HER3 signaling in breast cancer cells, and enhanced the responsiveness of human ER+ breast cancer tumors in mice to endoxifen, an active metabolite of TAM. Taken together, the results support PRCP as a potential prognostic marker for outcome in breast cancer patients and a novel target to improve endocrine therapy in ER+ breast cancers.

We found high PRCP expression is associated with reduced overall survival in ER+/Her2- breast cancer patients and earlier recurrence in ER+/Her2- breast cancer patients treated with endocrine therapy. This suggests high PRCP expression may be a marker for poor outcome and poor response to endocrine therapy in these patients. Indeed, cells with overexpression of PRCP show resistance to TAM or endoxifen and are sensitized to these treatments by PRCP inhibitor.

A question that arises is how PRCP promotes endocrine therapy resistance. The AKT-mTORC1 pathway is activated downstream of multiple RTKs and contributes to endocrine therapy resistance. Cells with overexpression of PRCP had heightened AKT-mTORC1 compared to parental MCF7 cells both basally and in response to TAM. High expression and activity of IGF1R and EGFR/HER2 promote TAM resistance and are associated with worse patient outcome in breast cancer.^14–18^ B6-9 cells with overexpression of PRCP displayed increased IGF1R and ErbB3 signaling and heightened AKT activation in response to heregulin (ErbB3 ligand) and insulin (IGF1R/IR ligand) compared to MCF7. Combined inhibition of IGF1R and HER3 sensitizes TRC cells to endoxifen. Moreover, analyses of clinical databases showed that PRCP expression correlates with IGF1/NRG1 expression and their target genes *EGR1, KLF2, CTGF*, and *ETV1*. High expression of *PRCP, IGF1, NRG1, EGR1*, KLF2, and *ETV1* also significantly correlates with reduced RFS of endocrine treated patients. Overall, the results support a model in which PRCP promotes endocrine therapy resistance by promoting IGF1R/HER3 signaling and subsequent activation of AKT. A remaining but unanswered question is how PRCP increases or promotes IGF1R/HER3 signaling. PRCP cleaves different GPCR agonist peptides such as angiotensin II and α-MSH, though the full repertoire of PRCP substrates is not established. There is abundant crosstalk between GPCR and RTK signaling pathways that regulate cancer cell proliferation. We recently reported that overexpression of PRCP in B6-9 cells increased cleavage of angiotensin II to angiotensin 1–7 that coincided with increased activation of PKA and CAMKII.^27^ Moreover, inhibition of PKA and CAMKII decreased EGF-induced activation of EGFR and AKT and heregulin-induced activation of Her3, IRS1, and AKT.^27^ Although more work is needed, we hypothesize PRCP substrate cleavage regulates or alters the cross-talk between GPCRs and RTKs, and in this way increases RTK signaling and subsequent endocrine therapy resistance.

The PRCP inhibitor PRCP-7414 (PRCPi) when given alone reduced growth of ER+ MCF7 cell line and PDX tumors in mice. As expected, this was associated with a reduced levels of phosphorylated (activated) AKT in tumor lysates. The results indicate PRCPi is bioavailable with anti-tumor activity and reduces activated AKT in tumors as it does in cells. TAM is metabolically activated to 4-OHT and endoxifen. Endoxifen alone also reduced growth of ER+ MCF7 cell line tumors and growth of ER+ PDX tumors in mice. Most importantly, combined PRCPi plus endoxifen caused the most pronounced reduction in tumor growth. These results support PRCP as a potential viable target to increase endocrine therapy responses in ER+ tumors, though side effects including blood pressure changes may occur. Future directions may include developing effective ways to inhibit PRCP while minimizing side effects.

## Conclusions

PRCP is an adverse prognostic marker and a potential therapeutic target to enhance endocrine therapy response in ER+ breast cancer patients.

## Materials and methods

### Cells and reagents

MCF7 breast cancer cell line was obtained from ATCC. MCF7 cells were grown in DMEM medium, with 10% fetal bovine serum (FBS), penicillin (100 U/mL) and streptomycin (100 µg/mL). Cells were plated 24 h before treatment with different drugs at the indicated concentrations.^27^ Recombinant human heregulin (HRG) and IGF1 were obtained from Sigma Chemical Co (St. Louis, MO).^27^ PRCP inhibitor (PRCP-7414); catalog number 504044 was from Calbiochem. OSI906 and lapatinib are obtained from Selleckchem.

### Immunoblotting

Immunoblotting was done as described previously.^27^ Whole cell extracts were prepared by scraping cells in lysis buffer (150 mM NaCl, 5 mM EDTA, 0.5% NP40, 50 mM Tris, pH 7.5), resolved by sodium dodecyl sulfate polyacrylamide gel electrophoresis (SDS-PAGE) and transferred to polyvinylidene difluoride membranes (Thermo Fisher Scientific). Antibodies to p-ErbB3 (Y1289), ErbB3, p-IGF1R (Y1135), IGF1R, p-AKT (S473), pan AKT, pmTOR (S2448), mTOR, pS6K (T389), S6K were from Cell Signaling; Phospho-IRS1 (Y612) was from EMD Millipore. IRS1 was from Bethyl Laboratories. PRCP antibody was from R&D systems. β-actin antibody was from Santa Cruz. Primary antibodies were detected with goat anti-mouse or goat anti-rabbit secondary antibodies conjugated to horseradish peroxidase (Life Technologies), using Clarity chemiluminescence (BIO-RAD). Each presented experiment was a representative of at least two repeatable experiments. (They may ask for all the catalog and lot numbers and dilutions used for the antibodies).

### Flow cytometry

For cell cycle analysis, cells were harvested and fixed in 25% ethanol overnight, as we described previously.^27^ The cells were then stained with propidium iodide (25 µg/ml, Calbiochem). Flow cytometry analysis was performed on a Gallios™ Flow Cytometer (Beckman Coulter), analyzed with FlowJo 10 (Treestar Inc). For each sample, 10,000 events were collected. Experiments are done in triplicate and repeated two or three times.

### Retroviral and lentiviral infection

Human PRCP cDNA in pFB-retroviral vector was co-transfected with packaging vector (pIK) using Fugene (Promega) into 293 FT cells to generate retroviral supernatants as described.^27^ The retroviral supernatants were collected 24 h after transfection and then used to infect subconfluent MCF7 cells as described.^8^

### Tissue microarray construction and immunohistochemistry

Tissue microarray construction and immunohistochemistry were as described previously^27^ Primary breast cancer tissues were acquired from archived formalin-fixed, paraffin-embedded (FFPE) pathology tissue blocks in the department of pathology at Rush University Medical Center. For an unbiased analysis, 400 patients consecutively treated from 2000 to 2005 at Rush University Medical Center that have complete follow-up records were selected, including all age and race groups. All the patients are female. For each case, a relevant area of interest was identified on hematoxylin and eosin (H&E) slides and marked along with the corresponding FFPE tissue blocks by two pathology residents in the Department of Pathology. Out of the 400 selected tissue blocks, only 197 cases (127 ER+/Her2 negative cases) contained enough intact tumor tissues for construction of the TMA. The 197 annotated tissues were submitted to the Pathology Tissue Microarray Core Lab at the University of Illinois at Chicago for tissue microarray (TMA) construction. Two representative cylindrical cores of 1.0 mm in diameter were taken from each donor block and re-embedded into recipient paraffin blocks using a TMA Master arrayer (3D Histech Ltd., Budapest, Hungary) following standard procedures^28^ In total, 394 breast cancer tissue cores and 24 de-identified morphologically benign breast tissue cores arranged as orientation markers were distributed onto four TMA blocks. To increase the adherence of the re-embedded tissue, the recipient blocks were incubated overnight at 37°C prior sectioning. Scientific analysis of the cohort of tumors was approved by the Institutional Review Boards at Rush University Medical Center.

The second TMA (TMA-05-7) contains 32 cases of primary ER+ breast cancer tumors from patients treated at Fox Chase Cancer Center that have complete follow-up records. The TMA was obtained from the Fox Chase Biosample Repository Facility under Material Transfer Agreement. The third TMA contains 66 primary tumors of breast cancer patients whose tumors recurred after surgical resection. The TMA was obtained through a collaboration with Dr. Elizabeth Wiley in the Department of Pathology at UIC.

The Proteinatlas validated anti-PRCP antibody (HPA017065) was acquired from SigmaAldrich. All the TMA samples were IHC stained with the PRCP antibodies with hematoxylin counterstain using standard procedures at UIC histology core facility. The IHC staining was interpreted by two pathologists in a blind way and PRCP positivity defined as tumors in which more than 60% of the tumor cells have moderate to strong staining pattern.

### Gene expression omnibus dataset analysis

GSE25066 dataset contains 290 ER+ breast cancer cases treated with endocrine therapy with recurrence free survival data available^21,22^ The dataset was analyzed using the Kaplan-Meier Plotter software and database (http://kmplot.com/analysis/). The auto select best cutoff option was used to divide patients into high vs. low expression of genes of interest. Kaplan-Meier survival curves were plotted to compare recurrence-free survival times between high vs. low expression of the three genes. Log-rank test was used to determine significance between groups.

### Analysis of gene correlation using the Tumor IMmune Estimation Resource (TIMER)

The TIMER website (https://cistrome.shinyapps.io/timer/) was used to analyze gene correlation in invasive breast cancer patients from the TCGA database. It contains 1093 cases of invasive breast cancer. Images and statistical analyses are automatically generated by the onsite software.

### In vivo xenografting and therapy

NOD.Cg-*Prkdc^scid^*/J (NOD scid) and NOD.Cg-*Prkdc^scid^Il2rg^tmlWjl^*/Sz (NOD-SCID IL2rγ^null^; NSG) mice were obtained from the Jackson Laboratory (Bar Harbor, ME, USA). The mice were maintained under specific pathogen-free conditions in accordance with the ethical guidelines for the care of these mice at the Comparative Research Center of Rush University Medical Center^27^ The mice were 6–8 weeks of age at the time of transplant. All mice were subcutaneously inoculated with estrogen pellets (1.5 mg/pellet, 90-day release) obtained from Innovative Research of America before xenografting.

For MCF7 cell transplantation, 10 million of disaggregated MCF7 cells (passage three from ATCC) were resuspended in 100 μl of a 1:1 v/v mixture of cold DMEM:Matrigel (BD Biosciences, San Jose, CA) and kept on ice until transplantation. Cells were subcutaneously injected into the left mammary fat pads of NOD scid mice using 23 G needles. When tumors reached the size of 300 mm^3^, the mice were randomly divided into 4 groups (5 mice/group) for treatment.

The ER+/PR+/ErbB2- PDX tumor (TM00386) was obtained from Jackson Laboratory. When the PDX tumor reached 1 cm^3^ volume, the tumor bearing mice were euthanized. Using aseptic technique, the tumor was removed and then minced into the smallest possible pieces using forceps and a scalpel. The minced tumors were transferred into a 1 ml syringe and then subcutaneously injected into 20 NSG mice (100 µl/mice) using a 14 G needle. When tumors reached 300 mm^3^, the mice were randomly divided into four groups (5 mice/group) for treatment.

For both MCF7 tumors and PDX tumors, the mice were treated with vehicle, endoxifen (20 mg/kg/day, 5 days/week), PRCPi (20 mg/kg/day, 5 days/week), or combination of endoxifen and PRCPi. Both endoxifen and PRCP were solubilized in Cremophor EL formulation for intraperitoneal injection. Tumor growth and body weight were then monitored with a caliper twice per week. When tumors reached 1 cm^3^ volume, the mice were euthanized. At necropsy, the tumors were harvested for further analysis.

### Statistical analysis

One-way analysis of variance (ANOVA) and Student’s *t*-test were used to determine the statistical significance of differences among experimental groups. Student’s *t*-test was used to determine the statistical significance between control and experimental groups.^27^

## Supplementary Material

Supplemental Material

## Data Availability

The invasive breast cancer dataset for analysis of gene correlation is available at The TIMER website (https://cistrome.shinyapps.io/timer/). The GSE25066 dataset was available at the Kaplan-Meier Plotter website (http://kmplot.com/analysis/).
