# Supplementary material for: Prolylcarboxypeptidase promotes IGF1R/HER3 signaling and is a potential target to improve endocrine therapy response in estrogen receptor positive breast cancer
Source: Cancer Biol Ther. 2022 Nov 4;23(1):1–10. doi: 10.1080/15384047.2022.2142008 (PMC9639567; doi:10.1080/15384047.2022.2142008)
Supplement: Supplemental Material [file KCBT_A_2142008_SM9819.zip › Supplementary_Data_revision.docx]

**Supplementary Data**

**Figure S1. PRCPi sensitizes tumors to endoxifen.** A and B. This is an extension of data already presented in Figure 5. MCF7 tumors and ER+ PDX tumors were treated with vehicle. Endoxifen, PRCPi, or combination for the indicated times. Average tumor volumes (mm^3^) in each group were plotted with SE indicated. There are significant differences between vehicle and endoxifen or PRCPi (p<0.05) in both tumors. There are significant differences between single drug and combination therapies (p<0.05) in both tumors. C and D. Average mouse body weights in each group are presented with SD indicated. There are no differences between groups (p˃0.05).
